# The current status of the case report: Terminal or viable?

**DOI:** 10.2349/biij.5.1.e4

**Published:** 2009-01-01

**Authors:** NH Abu Kasim, BJJ Abdullah, J Manikam

**Affiliations:** 1 Department of Conservative Dentistry, Faculty of Dentistry, University of Malaya, Kuala Lumpur, Malaysia; 2 Department of Internal Medicine, Faculty of Medicine, University of Malaya, Kuala Lumpur, Malaysia; 3 Department of Biomedical Imaging, Faculty of Medicine, University of Malaya, Kuala Lumpur, Malaysia

**Keywords:** Case reports, relevance

## Abstract

The case report, which has a long history in medicine, has seen its fortune wax and wane with time. We discuss the challenges facing the continued survival of the case report, including the inability of journals to cope with the increased load and increased cost of publication, ethical issues, the impact factor and the rise of evidence-based medicine. We highlight the important role that the case report will continue to play in medical research and education, as a means of sharing information and detecting novelty through observations. Most importantly, the case report serves as a stepping stone for young physicians and practitioners into the world of medical writing.

## CASE REPORT

Following a motor vehicle accident, an unknown patient was brought by passers-by to the trauma department. On admission, the patient was disheveled, unconscious (GCS of 8/15) with lacerations to his scalp, bruising to the chest and fracture of his left femur. He was not moving his lower limbs. Following intubation and resuscitation with IV fluids, the patient was sent for a CT of the head, spine, chest, abdomen and pelvis. There were severe cerebral contusions with oedema, no shift of midline or evidence of any focal parenchymal bleed. A comminuted fracture of the L4 vertebral body with narrowing of the spinal canal was noted. The chest showed lung contusions while the abdomen did not show any evidence of injury. The great vessels were normal.

Patient was subsequently found to be a Mr. Case Report (CR). CR was once a very respectable part of the medical research establishment and was given recognition and space in most medical publications. However, over the years, due to increased competition, changes in the way his contribution was being assessed, as well as the increasing commercialisation of medical publications, CR slowly became sidelined and withdrawn from the readers. At times, CR was even told not to show his face around the more important original research reports (ORR), the reviews (R) or even the invited commentaries (IC). CR went into depression as CR had lost its position in society. The community was unaware of CR’s plight and there were few who would stand up for CR’s role and importance. CR become a vagabond and would live in the streets begging for food. There were two occasions on which CR attempted suicide but this was lost on his old friends, the ORR, R and IC who were too busy being important. This current admission to the trauma department was the result of CR jumping off a bridge, then being hit by an oncoming car.

After decompression and fixation of his spine, CR made a slow recovery with support from some of his old trusted friends who nursed him back to health. However, CR never recovered to full function. CR is still no longer in the limelight but CR is having a new life by being shown in specialty medical publications who feel that CR must continue to play his role.

## DISCUSSION

History repeats itself; that’s one of the things that’s wrong with the history

– Clarence Darrow

Seminal early medical texts such as the Chinese Yellow Emperor’s Classic, the Ancient Egyptian Smith Papyrus, and Hippocrates’ Aphorisms, all expounded methods based on the learning from the authors’ personal cases. Case reports have provided a rich resource for teaching and research in medicine.

A case report is a detailed report of the symptoms, signs, diagnosis, investigative results, treatment, complications of treatment, and follow-up of an individual patient. Case reports [1] are a brief description of a case with unique feature/s not previously reported, e.g.

- A positional or quantitative variation of the anatomical structures.
- A previously unreported clinical condition
- An unexpected association between diseases or symptoms.
- A unique or rare features observed while imaging recognised disease or lesion;
- A unique therapeutic or interventional technique
- A complication of a radiological procedure or treatment.

Research, which has become an integral part of medical careers, is based on evidence-based medicine, on the one hand, and genomics on the other. Evidence-based medicine establishes a strict hierarchy, with CRs being referred to as anecdotal findings. The CR has been relegated as the lowest form of medical research with meta-analysis and randomised clinical trials (placebo-controlled, double-blind studies) being clearly preferred. Yet, the CR formed an important part of early medical journals long before editors worshipped at the altar of evidence-based medicine [2].

For example due to the large backlog of CR, going back several years in some instances, some journals have resorted to temporary moratoriums on case report submissions, until some predetermined date, while more stringent criteria are being established for accepting future case reports. Others have decided to only publish abstracts online, with the full content either on the journal or on a professional body's website. There are also now statements that electronic versions will have faster turn-around than hard copy submissions. The CR is often deemed to be of lower priority by the referees, regardless of the impact factor of the journal, which is partly to blame for the delay in processing the submissions.

Journals that do continue to publish CRs are receiving more of them and thus, have developed new and more stringent guidelines. A random search of Pubmed listed 88,516 CR till July 2008, 181,731 case reports in 2007 and 405,317 in 2006 compared to 74,266 in year 2000. 13.5% (183,349 of 1,355,539) of all the references in the 120 core clinical journals1 Abridged Index Medicus (AIM) journal titles (www.nlm.nih.gov/bsd/aim.html) (accessed Oct 16, 2001). are case reports [3]. Therefore if one desires to see his or her manuscript grace the pages of a peer-reviewed journal, it needs to be of high quality [4].

When it comes to imaging journals, CRs have been removed completely from some journals e.g. AJR Integrative Imaging Supplement [5], and are submitted as a Teaching File instead [6]. CRs are published as a supplement in others or only available in the online version. Those who continue to treat it as a vital component of medical education, research and publication, do accept and publish case reports. There is now even a Journal of Radiological Case Reports (www.radiologycases.com) which has started to accept case reports.

Journals that continue to accept CRs show varying acceptance in terms of submission requirements of case reports. Most generally limit the length, although the required page length varies [7-9]. The number of figures is also generally stated. Increasingly, imaging journals are beginning to accept streaming videos with specific formats for the Multimedia file or even allowing interactive datasets which allow the reader to manipulate and view the pathology for themselves.

Most journals require the following order of presentation:

- Abstract,
- Keywords,
- Introduction,
- Case Report, and
- Discussion,
- Followed by references, tables, and legends.

Why has the CR lost its place in an increasing number of journals? The opponents’ state that:

- A CR is a form of anecdotal evidence; it is therefore less scientifically rigorous than controlled clinical data involving a larger sample size.
- The CR has a low level of general application to the practice of evidence-based care since case reports have certain inherent limitations [10-12]
- Many CRs are submitted more in the hope of padding out a thin Curriculum Vitae for career progress rather than with any intention of expanding scientific knowledge.
- The rampant abuse of gift authorships [13] is against the ethics of scientific publication.

However, the decreased scientific rigour of the CR may be due to the failure of the journal/editors/reviewers to evaluate the preparation and the accuracy of the reports more stringently [14]. Thus journals need not only ensure strict adherence to concise CRs that provide clear educational value to the clinician, but also assist the authors with constructive criticisms on how their submissions can be enhanced. It is therefore vitally important for journals to publish clear guidelines for authors who plan to write a case report. The guidelines should specify the length of the manuscript, number of words, number of figures, and number of references. This also helps reviewers when reviewing the manuscripts, providing constructive criticism, and suggesting revisions. It may also be helpful to require authors to explain in their covering letter/electronic submission exactly why they think that their CR is of sufficient interest to be considered for publication. All these requirements will eventually benefit the readers as the journal is able to provide concise and focused reports that include useful information.

Proponents argue that case reports have value within the scientific method since they serve as valuable tools for sharing information. There are several means by which clinicians and scientists communicate with one another, and these methods of communication can often help determine the kind of format in which specific forms of information can be shared. For example, scientists communicate to other scientists via bench research reports, while clinicians communicate to other clinicians through literature reviews, i.e. they provide information on all that might be known about a given topic. And clinicians communicate to scientists through case reports [15].

The CR “…..permits discovery of new diseases and unexpected effects (adverse or beneficial) as well as the study of mechanisms and play an important role in medical education. Case reports and series have a high sensitivity for detecting novelty and therefore remain one of the cornerstones of medical progress; they provide many new ideas in medicine” [1]. Scientific observations in a single case may not prove anything in themselves, but when presented to a wider audience may trigger larger and more significant studies [2]. It must also be recognised that medical knowledge has been traditionally built case by case. If we acknowledge the limitations of the case report we will not need to neglect its importance in our search for solid evidence [16].

In 1985, the American Medical Association reprinted 51 papers from the Journal of the American Medical Association that had significantly changed the science and practice of medicine during the 150 years of the organisation’s existence. Interestingly, 5 of these papers were case reports [17]. Famous authors of scientific case reports which have been the basis of progress of medicine have included:

- Sir William Osler, himself the author of many such scientific observations, encouraged other physicians to “Always note and record the unusual . . . When you have made and recorded the unusual or original observation . . . publish it.”
- Sigmund Freud reported on numerous cases.
- Frederick Treves reported on "The Elephant Man".
- Paul Broca reported on language impairment following left hemisphere lesions in the 1860s.
- Joseph Jules Dejerine reported on a case of pure alexia.
- William MacIntyre reported on a case of multiple myeloma (described in the 1840s).
- The case report on AIDS and Kaposi's sarcoma.
- German psychiatrist Alois Alzheimer first described Alzheimer's disease by in 1906 [18].

We are all aware of how we started in medical writing – it was probably the CR that introduced the uninitiated young physicians and private practitioners into the research world who otherwise would not have had access to academic clinical or basic science research. If the CR gets published, it becomes an asset in the CV of the young doctor which then gives him or her the pride and courage to proceed along the subsequent phases of medical research. The CR serves as a “safe” rite of passage without placing too much at stake, while enabling the development of thought processes geared towards research. As we all recognise, any kind of research, including writing CRs, involves a lot of hard work and persistence, which is good training for the future of our careers.

We have also had numerous instances where images from case reports have been used to enhance the quality of teaching material e.g. textbooks, presentations, etc. Case reports can also entertain and interest, leavening worthy but dry pages of statistical analysis or molecular methods with some real clinical medicine [2]. Lastly, the act of writing a report provides an opportunity for one to practise concisely written communication, to learn about a topic, and to think critically [10,13].

One may wonder why case reports are so popular. Is it because humans are natural storytellers and case reports are a form of “short” stories? We have been telling stories for the last 30,000 years. However, about 2,500 years ago, thanks to Plato, Socrates and other ancient Greeks, Western thinking switched to the mode of inquiry, dialogue, argument and reason when there was knowledge to impart. While scientific insight and technological breakthroughs may dominate the practice and teaching of medicine, good old-fashioned storytelling is increasingly seen as vital in shedding light on the human side of medicine. With narrative competence, physicians can reach out and join their patients in illness, recognise their own personal journeys through medicine, acknowledge kinship with, and duties toward, other health care professionals, and inaugurate consequential discourse with the public about health care [19]. Narrative medicine is proposed as a model for humane and effective medical practice.

The economics of publishing, the logistics of the peer-review, the increased competition between journals, the introduction of ranking systems e.g. impact factor, as well as other factors have been partly responsible for the downgrading of the CR. Authors prefer to submit their best work to journals with the highest impact factors, and these are derived from a calculation which includes the number of citations made to articles in the journal. In terms of attracting citations, case reports tend to be at the bottom of the pile, and therefore it may not be in the long-term interest of any ambitious biomedical journal to include them in the running order [2]. The limited page space within a journal therefore tends to be dedicated to experimental studies which have a higher effect on the journal's impact factor, unlike the diminutive effect of case reports [20]. With the increased cost of hard copy publication, the numbers of pages that can be printed are limited, and the journals have to make a choice of which categories to include in hard copy.

Will the case report continue to be relevant? Our answer is an emphatic 'yes', especially with the advent of electronic publishing which has both reduced the cost and increased the speed of sharing information. Along with its interactive potential, the electronic CR has vast possibilities for teaching and learning. The trend towards electronic medical publications is evident as the younger generation is very comfortable with the Internet. We believe that this generation of young researchers will derive great benefit from CRs and be driving even higher levels of CR publications. If the novices can be convinced that the CRs are important, then there is a very strong possibility of markedly increasing the numbers of future researchers and writers.

“……..And besides, if an ectopic pregnancy does ever occur in the tongue, I would rather read about it (and believe it) in a well-documented article in one of our respected journals than in a supermarket checkout magazine.” [21].
